# Duration of dual antiplatelet therapy following percutaneous coronary intervention on re-hospitalization for acute coronary syndrome

**DOI:** 10.1186/1471-2261-14-21

**Published:** 2014-02-18

**Authors:** Shih-Chin Chen, Fei-Yuan Hsiao, Chii-Ming Lee, William Wei-Yuan Hsu, Churn-Shiouh Gau

**Affiliations:** 1 Graduate Institute of Clinical Pharmacy, College of Medicine, National Taiwan University, 33, Linsen South Road, Taipei, Taiwan; 2 School of Pharmacy, College of Medicine, National Taiwan University, Taipei, Taiwan; 3 Department of Pharmacy, National Taiwan University Hospital, Taipei, Taiwan; 4 Department of Internal Medicine, College of Medicine, National Taiwan University, Taipei, Taiwan; 5 Department of Computer Science and Engineering, National Taiwan Ocean University, Keelung, Taiwan; 6 Institute of Information Science, Academia Sinica, Taipei, Taiwan; 7 Center for Drug Evaluation, 3F, No. 465, Zhongxiao E. Rd. Sec. 6, Taipei, Taiwan; 8 Food and Drug Administration, Ministry of Health and Welfare, Taipei, Taiwan

**Keywords:** Acute coronary syndrome (ACS), Percutaneous coronary intervention (PCI), Clopidogrel, Dual antiplatelet therapy, Drug eluting stent (DES)

## Abstract

**Background:**

The optimal duration of dual antiplatelet therapy after percutaneous coronary intervention (PCI) remains uncertain. The objective of this study was to examine the association between duration of dual antiplatelet therapy and re-hospitalization for acute coronary syndrome (ACS) in ACS patients who underwent PCI.

**Methods:**

We identified 975 newly diagnosed ACS patients who underwent PCI between July, 2007 and June, 2009, at a medical center in Taiwan. Cox proportional hazard models were used to examine the association between duration of dual antiplatelet therapy (9 months, 12 months and 15 months) and risks of re-hospitalization for ACS.

**Results:**

At a mean follow-up of 2.3 years, we found that use of clopidogrel for ≥ 12 months was associated with a decreased risk of re-hospitalization for ACS (adjusted HR 0.59, 95% CI 0.36-0.95; p = 0.03). However, use of clopidogrel for ≥ 15 months was not associated with a decreased risk of re-hospitalization for ACS (adjusted HR 0.57, 95% CI 0.29-1.13; p = 0.11). Similar results were found in patients who implanted drug-eluting stents (DES), for whom at least 12 months of clopidogrel therapy is especially critical.

**Conclusion:**

The benefit of ≥ 12 months of clopidogrel use in reducing the risk of re-hospitalization for ACS was significant among ACS patients who underwent PCI and was especially critical for those who implanted DES.

## Background

The clinical benefits of clopidogrel in combination with aspirin in acute coronary syndrome (ACS) patients undergoing percutaneous coronary intervention (PCI) have been well demonstrated [1-4]. However, the optimal treatment duration of dual antiplatelet therapy following PCI remains uncertain. Findings from the PCI subpopulation of the Clopidogrel in Unstable Angina to Prevent Recurrent Events (PCI-CURE) [3] trial suggested that 3-12 months duration of clopidogrel treatment after PCI significantly reduced the risk of cardiovascular death, myocardial infarction and stroke. Nevertheless, other studies showed no benefits from continuing clopidogrel beyond 6 months [5]. The 2011 guideline issued by the American College of Cardiology (ACC) and the American Heart Association (AHA), however, has advocated an at least 12 months of dual antiplatelet therapy following PCI and an extended duration of treatment to at least 15 months and possibly indefinitely for patients who received drug-eluting stents (DES) [6]. The European Society of Cardiology (ESC) Guidelines for the management of ACS in patients presenting without persistent ST-segment elevation and ST-elevation myocardial infarction (STEMI) also suggest a 12-months of dual antiplatelet therapy for these patients [7,8].

Several issues have been raised regarding the latest guidelines [6]. First, the extended duration of 15 months was suggested mainly due to the concern of late stent thrombosis associated with DES. Although a slight increase in the risk of DES thrombosis after the first year has been noted [9,10], there is no report to support the effectiveness of a 15-months clopidogrel therapy. Secondly, prolonged clopidogrel therapy could be costly and associated with risks for major bleeding [11]. Thirdly, the recommendation of 12-months or even15-months of dual antiplatelet therapy may not be generalizable to the non-US ACS population, due to regional and health insurance plan limitations. The conflict between the reimbursement policy and latest clinical guidelines may further reflect urgent needs for more evidence on the minimal necessary duration of dual antiplatelet therapy for ACS patients.

The objective of this study was to examine the association between duration of dual antiplatelet therapy and re-hospitalization for ACS in ACS patients who underwent PCI. Specifically, we evaluated the clinical benefit of at least 15-months of dual antiplatelet therapy among the DES population.

## Methods

### Patient population

Patients included in this retrospective cohort study were those who hospitalized for a newly-diagnosed ACS (*International Classification of Disease, Ninth Revision*, Clinical Diagnosis (ICD-9) CM codes: 410.xx, 411.xx, and 414.xx) and who underwent PCI between July 1, 2007 and June 30, 2009 at a 2000-bed, university-affiliated, medical centre in Taiwan. Eligible patients were those with no documented ACS-related diagnoses or procedures before the index ACS hospitalization. Patients who underwent both PCI and coronary artery bypass graft (CABG), who received fibrinolytic agents during the index ACS hospitalization, who received clopidogrel less than 7 days after discharge, who lost to follow-up or for whom follow-up data were not available were excluded.

### Data collection

Baseline data were obtained from the hospital computerized medical system and confirmed by reviewing patients’ medical records. For each patient, data during the index ACS hospitalization were collected, including demographic characteristics, family history, smoking history, body mass index, biochemistry tests (including serum creatinine, total cholesterol, triglyceride, fasting blood glucose, hemoglobin A1c), comorbidities, left ventricular ejection fraction, angiographic findings including extent of coronary artery stenosis, stent type, diameter, length, and site of stent deployment. Additional data collected were concomitant medications used during the follow-up.

### Clopidogrel use

All clopidogrel prescriptions were obtained from the hospital electronic pharmacy prescription database. The numbers of refills, doses, and pills dispensed of each prescription after discharge were identified to calculate the duration of clopidogrel use.

### Study endpoint

The study endpoint was a re-hospitalization for ACS, which was defined as a subsequent hospitalization with a primary diagnosis of ACS (ICD-9 CM codes: 410.xx, 411.xx, 414.xx, and 996.72) after discharge from the index ACS hospitalization. Medical records were reviewed to further confirm the subsequent ACS hospitalization.

### Study groups

In order to explore the association between clopidogrel duration and re-hospitalization for ACS, we compared the study endpoint in patients who received clopidogrel for a certain duration after discharging from the index hospitalization (categorized as “continuous users”) with those who did not (categorized as “discontinuous users”). Three predefined time points were used in our study: 9 months, 12 months and 15 months of clopidogrel use. For example, in the “9-months analysis”, patients who were event-free at 9 months were categorized into continuous users (received clopidogrel for ≥ 9 months) and discontinuous users (received clopidogrel for < 9 months). On categorization, a window of 30 days was allowed for potential time lag between 2 prescriptions. Similar algorithm were applied to the 12- and 15- months time points.

### Statistical analysis

Descriptive analyses of the differences between “continuous users” and “discontinuous users” were conducted using χ^2^ and F values for categorical variables and t-tests for continuous variables.

Unadjusted event rates were compared between the continuous and discontinuous clopidogrel users. Cox proportional-hazard models were performed to estimate the association between duration of clopidogrel use and the risk of re-hospitalization for ACS. Patients who died during the follow-up were censored. The univariate relations between baseline variables with ACS re-hospitalization were first performed. We then performed multivariable Cox regression models adjusted for variables with a significance level of p-value < 0.15 identified in the univariate analyses. Stepwise selections were further used to determine variables to retain in the models. The variable denoting duration of clopidogrel use was retained in the model irrespective of its statistical significance. Adjusted hazard ratios (HRs) and 95% confidence intervals (CIs) were estimated based on the stepwise selection. We performed similar analyses in patients who implanted DES during the index hospitalization. All analyses were performed using the SAS statistical software, version 9.1 (SAS Institute, Cary, North Carolina). This study was approved by the Institutional Review Board of National Taiwan University Hospital (200911017R).

## Results

**Figure 1:**
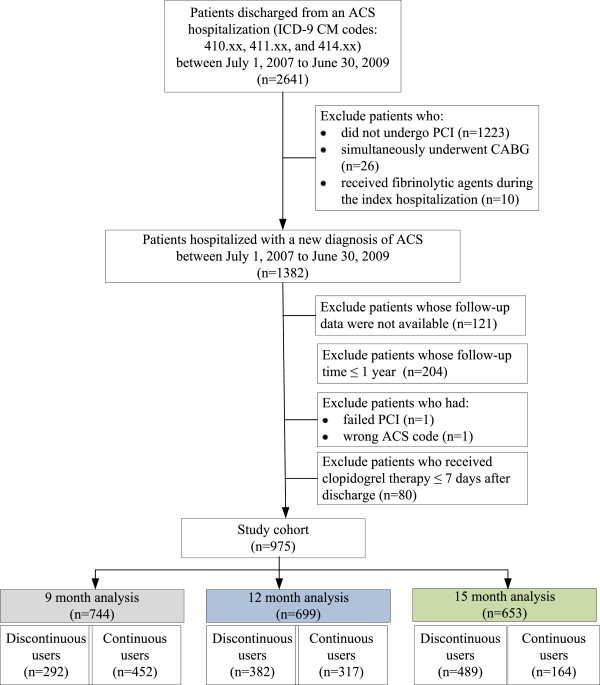
Study flowchart.

Of the 2,641 patients newly hospitalized for ACS between July 1, 2007 and June 30, 2009, we identified 1,382 patients who underwent PCI. After applying the exclusion criteria, we identified 975 patients as our initial study cohort (Figure 1).

### The 9-months analysis

The 9-months analysis included 744 patients who were event-free at 9 months after the index hospitalization. Patients in the continuous and discontinuous groups were well balanced in terms of most baseline characteristics (Tables 1 and 2). At a mean follow-up of 815 days (27 months), the cumulative rates of ACS re-hospitalization were 14.6% and 20.9% for continuous and discontinuous clopidogrel users, respectively (Table 3). The clinical benefit of use of clopidogrel for ≥ 9 months in reducing hazards of ACS re-hospitalization was non-statistically significant (adjusted HR 0.69, 95% CI 0.48-1.00; p = 0.05) (Table 3).

**Table 1:** Baseline characteristics of study patients; by clopidogrel duration (9, 12, 15 months) analysis

	**9-months**			**12-months**			**15-months**		
	**Discontinuous users (n = 292)**	**Continuous users (n = 452)**	***p*** *	**Discontinuous users (n = 382)**	**Continuous users (n = 317)**	***p*** *	**Discontinuous users (n = 489)**	**Continuous users (n = 164)**	***p*** *
Male	237 (81.2%)	350 (77.4%)	0.22	306 (80.1%)	248 (78.2%)	0.54	386 (78.9%)	131 (79.9%)	0.79
Age (years)	61.8 ± 12.0	63.1 ± 11.9	0.15	62.0 ± 12.3	63.3 ± 11.9	0.17	61.8 ± 11.7	64.2 ± 12.8	0.03*
BMI (kg/m^2^)	25.7 ± 3.8	25.5 ± 3.6	0.52	25.6 ± 3.7	25.6 ± 3.6	0.97	25.6 ± 3.7	25.7 ± 3.5	0.70
Current smokers	102 (35.1%)	128 (28.3%)	0.05	131 (34.3%)	90 (28.4%)	0.09	162 (33.1%)	43 (26.2%)	0.10
Previous smokers	56 (19.2%)	84 (18.6%)	0.82	72 (18.9%)	57(18.0 %)	0.77	91 (18.6%)	30 (18.3%)	0.93
Family history of CAD	61 (20.9%)	106 (23.5%)	0.41	78 (20.4%)	75 (23.7%)	0.30	105 (21.5%)	38 (23.2%)	0.65
Clcr (mL/min)	70.4 ± 25.4	68.8 ± 28.3	0.44	70.2 ± 26.1	68.6 ± 28.3	0.46	70.4 ± 26.2	66.9 ± 29.5	0.17
Cholesterol (mg/dL)	180.8 ± 39.4	182.4 ± 39.9	0.60	181.1 ± 37.2	182.8 ± 42.4	0.60	181.8 ± 38.8	181.9 ± 40.8	0.98
TG (mg/dL)	155.0 ± 98.4	146.2 ± 122	0.29	149.1 ± 95.0	150.0 ± 135	0.92	149.4 ± 118.8	149.5 ± 102	0.99
Glucose_AC (mg/dL)	113.2 ± 37.0	114.7 ± 34.9	0.63	113.1 ± 34.9	115.2 ± 37.8	0.52	114.7 ± 37.6	111.9 ± 33.2	0.46
Hb_A1c_ (%)	7.1 ± 2.2	7.0 ± 2.2	0.89	7.1 ± 2.2	6.7 ± 1.9	0.28	7.01 ± 2.0	6.48 ± 3.0	0.25
LVEF (%)	57.3 ± 11.4	55.9 ± 8.5	0.35	56.5 ± 10.6	55.9 ± 8.7	0.68	56.4 ± 10.0	55.1 ± 7.4	0.44
Comorbidities									
Diabetes mellitus (DM)	89 (30.5%)	143 (31.6%)	0.74	117 (30.6%)	100 (31.6%)	0.79	152 (31.1%)	54 (32.9%)	0.66
Uncontrolled DM	32 (11.0%)	65 (14.4%)	0.18	48 (12.6%)	43 (13.6%)	0.70	65 (13.3%)	20 (12.2%)	0.72
Hyperlipidemia	130 (44.5%)	219 (48.5%)	0.29	175 (45.8%)	154 (48.6%)	0.47	229 (46.8%)	79 (48.2%)	0.77
Hypertension	188 (64.6%)	314 (69.6%)	0.15	241 (63.3%)	223 (70.6%)	0.04*	316 (64.9%)	119 (72.6%)	0.07
Heart failure	14 (4.8%)	21 (4.7%)	0.93	21 (5.5%)	12 (3.8%)	0.29	19 (3.9%)	10 (6.1%)	0.23
Arrhythmia	36 (12.3%)	70 (15.5%)	0.23	49 (12.9%)	49 (15.5%)	0.32	66 (13.5%)	27 (16.5%)	0.35
Cerebrovascular disease	18 (6.2)	21 (4.7)	0.37	21 (5.5%)	15 (4.8%)	0.66	26 (5.3)	9 (5.5)	1.00
Peripheral vascular disease	7 (2.4)	3 (0.7)	0.05	5 (1.3%)	3 (1.0%)	0.65	6 (1.2)	2 (1.2)	1.00
Chronic kidney disease	12 (4.1%)	26 (5.8%)	0.32	16 (4.2%)	20 (6.3%)	0.21	24 (4.9%)	9 (5.5%)	0.84
End-stage renal disease	5 (1.7%)	7 (1.6%)	1.00	6 (1.6%)	4 (1.3%)	0.73	6 (1.2%)	4 (2.4%)	0.28
GI ulcer/bleeding	32 (11.0%)	55 (12.2%)	0.62	44 (11.5%)	41 (12.9%)	0.57	52 (10.6%)	26 (15.9%)	0.07
Anemia	9 (3.1%)	15 (3.3%)	0.86	13 (3.4%)	9 (2.8%)	0.67	19 (3.9%)	3 (1.8%)	0.21
Co-medication									
Aspirin	254 (87.0)	365 (80.8)	0.03*	334 (87.4)	229 (72.2)	<.0001*	433 (88.6)	88 (53.7)	<.0001*
ACEI/ ARB	188 (64.4)	310 (68.6)	0.23	239 (62.6)	220 (69.4)	0.06	313 (64.0)	110 (67.1)	0.48
β-blocker	187 (64.0)	319 (70.6)	0.06	233 (61.0)	221 (69.7)	0.02*	303 (62.0)	116 (70.7)	0.04*
Lipid-lowering agents	189 (64.7)	326 (72.1)	0.03*	251 (65.7)	223 (70.4)	0.19	319 (65.2)	118 (72.0)	0.11
Statin	177 (60.6)	313 (69.3)	0.02*	239 (62.6)	212 (66.9)	0.24	306 (62.6)	110 (67.1)	0.30
Warfarin	6 (2.1)	9 (2.0)	1.00	5 (1.3)	8 (2.5)	0.27	8 (1.6)	3 (1.8)	1.00
CCB	134 (45.9)	198 (43.8)	0.58	164 (42.9)	136 (42.9)	0.99	197 (40.3)	75 (45.7)	0.22
Nitrate	140 (48.0)	219 (48.5)	0.89	163 (42.7)	148 (46.7)	0.29	195 (39.9)	67 (40.9)	0.83
NSAID	28 (9.6)	40 (8.9)	0.73	33 (8.6)	21 (6.6)	0.32	29 (5.9)	14 (8.5)	0.24
COX-2 inhibitor	20 (6.9)	22 (4.9)	0.25	24 (6.3)	11 (3.5)	0.09	19 (3.9)	7 (4.3)	0.82
Digoxin	12 (4.1)	14 (3.1)	0.46	15 (3.9)	10 (3.2)	0.58	15 (3.1)	7 (4.3)	0.46
Diuretics	71 (24.3)	117 (25.9)	0.63	93 (24.4)	72 (22.7)	0.61	103 (21.1)	36 (22.0)	0.81

*p-value <0.05.

BMI = body mass index, CAD = coronary artery disease, Cl_cr_ = creatinine clearance, TG = triglyceride, Glucose_AC = fasting blood glucose, Hb_A1C_ = glycosylated hemoglobin, LVEF = left ventricular ejection fraction, DM = diabetes mellitus, GI = gastro-intestinal, ACEI = angiotensin-converting enzyme inhibitor, ARB = angiotensin II receptor blocker, CCB = calcium channel blocker, NSAID = nonsteroidal anti-inflammatory drug, COX = cyclooxygenase.

**Table 2:** Index hospitalization characteristics of study patients; by clopidogrel duration (9, 12, 15 months) analysis

	**9-months**			**12-months**			**15-months**		
	**Discontinuous users (n = 292)**	**Continuous users (n = 452)**	***p*** *	**Discontinuous users (n = 382)**	**Continuous users (n = 317)**	***p*** *	**Discontinuous users (n = 489)**	**Continuous users (n = 164)**	***p*** *
STEMI	60 (20.6%)	124 (27.4%)	0.03*	95 (24.9%)	80 (25.2%)	0.91	127 (26.0%)	35 (21.3%)	0.23
NSTEMI	22 (7.5%)	76 (16.8%)	<0.001*	41 (10.7%)	52 (16.4%)	0.03*	62 (12.7%)	26 (15.9%)	0.30
Non-elective PCI	43 (14.7%)	91 (20.1%)	0.06	69 (18.1%)	57 (18.0%)	0.98	89 (18.2%)	27 (16.5%)	0.61
IABP use	3 (1.0%)	8 (1.8%)	0.54	4 (1.1%)	7 (2.2%)	0.22	7 (1.4%)	3 (1.8%)	0.72
GP IIb IIIa inhibitors	7 (2.4%)	15 (3.3%)	0.47	12 (3.1%)	10 (3.2%)	0.99	15 (3.1%)	7 (4.3%)	0.19
Number of diseased vessels	1.90 ± 0.85	2.03 ± 0.85	0.04*	1.91 ± 0.86	2.06 ± 0.85	0.02*	1.93 ± 0.86	2.04 ± 0.84	0.19
Multivessel PCI	93 (31.9%)	157 (34.7%)	0.42	115 (30.1%)	119 (37.5%)	0.04*	151 (30.9%)	68 (41.5%)	0.01*
Total occlusion	76 (26.0%)	153 (33.9%)	0.02*	106 (27.8%)	108 (34.1%)	0.07	142 (29.0%)	56 (34.2%)	0.22
Ostium lesion	18 (6.2%)	42 (9.3%)	0.13	24 (6.3%)	28 (8.8%)	0.20	29 (5.9%)	19 (11.6%)	0.01*
Bifurcation lesion	11 (3.8%)	25 (5.5%)	0.27	12 (3.1%)	18 (5.7%)	0.10	17 (3.5%)	10 (6.1%)	0.14
Location of stent									
RCA	92 (31.5%)	162 (35.8%)	0.22	118 (30.9%)	124 (39.1%)	0.02*	161 (32.9%)	61 (37.2%)	0.32
LAD	177 (60.6%)	281 (62.2%)	0.67	238 (62.3%)	195 (61.5%)	0.83	303 (62.0%)	104 (63.4%)	0.74
LCX	78 (26.7%)	128 (28.3%)	0.63	99 (25.9%)	91 (28.7%)	0.41	131 (26.8%)	52 (31.7%)	0.22
LM	12 (4.1%)	27 (6.0%)	0.27	13 (3.4%)	19 (6.0%)	0.10	18 (3.7%)	12 (7.3%)	0.05*
Number of stents									
1 stent	159 (54.5%)	219 (48.5%)	0.11	206 (53.9%)	147 (46.4%)	0.08	261 (53.4%)	67 (40.9%)	0.02*
≥ 2 stents	122 (41.8%)	222 (49.1%)		163 (42.7%)	162 (51.1%)		215 (44.0%)	91 (55.5%)	
At least 1 DES	185 (63.4%)	369 (81.6%)	<0.001*	252 (66.0%)	271 (85.5%)	<0.001*	350 (71.6%)	140 (85.4%)	<0.001*
Paclitaxel	53 (18.2)	103 (22.8)	0.13	72 (18.9)	75 (23.7)	0.12	91 (18.6)	44 (26.8)	0.02*
Sirolimus	76 (26.0)	134 (29.7)	0.28	103 (27.0)	94 (29.7)	0.43	139 (28.4)	49 (29.9)	0.72
Zotarolimus	49 (16.8)	105 (23.2)	0.03*	65 (17.0)	84 (26.5)	0.002*	95 (19.4)	43 (26.2)	0.07
Everolimus	12 (4.1)	45 (10.0)	0.003*	19 (5.0)	35 (11.0)	0.003*	35 (7.2)	14 (8.5)	0.56
EPC-capturing	11 (3.8)	13 (2.9)	0.50	15 (3.9)	7 (2.2)	0.20	17 (3.5)	4 (2.4)	0.62
Total stent length (mm)	39.6 ± 25.1	45.5 ± 30.2	0.01*	39.7 ± 24.9	46.9 ± 31.1	0.001*	41.4 ± 26.8	49.0 ± 32.7	0.01*
Min. stent diameter (mm)	3.04 ± 0.43	2.96 ± 0.47	0.03*	3.03 ± 0.44	2.95 ± 0.46	0.03*	3.0 ± 0.4	3.0 ± 0.5	0.27

*p-value <0.05.

STEMI = ST-elevation myocardial infarction, NSTEMI = non ST-elevation myocardial infarction, PCI = percutaneous coronary intervention, IABP = intra-aortic balloon pumping, RCA = right coronary artery, LAD = left anterior descending, LCX left circumflex, LM = left main, EPC = endothelial progenitor cell.

**Table 3:** Adjusted hazards for ACS re-hospitalization by clopidogrel duration (9, 12, 15 months); full cohort

**Clopidogrel use**	**N**	**Cases**	**Crude rate**	**Unadjusted hazard ratio**	***p*** *	**Adjusted hazard ratio**	***p*** *
				**HR (95% CI)**		**HR (95% CI)**	
**9 months**							
Discontinuous	292	61	20.9%	1.00 (Reference)		1.00 (Reference)	
Continuous	452	66	14.6%	0.70 (0.49-0.99)	0.04	0.69 (0.48-1.00) ^*^	0.05
**12 months**							
Discontinuous	382	53	13.9%	1.00 (Reference)		1.00 (Reference)	
Continuous	317	29	9.2%	0.66 (0.42-1.04)	0.07	0.59 (0.36-0.95) †	0.03
**15 months**							
Discontinuous	489	45	9.2%	1.00 (Reference)		1.00 (Reference)	
Continuous	164	13	7.9%	0.84 (0.45-1.56)	0.58	0.57 (0.29-1.13) ‡	0.11

HR = hazard ratio, CI = confidence interval.

^*^Adjusted for comorbidities (hypertension, DM uncontrolled, myocardial infarction), use of aspirin, multivessel PCI, number of stents, at least 1 DES, total stent length > 45 mm, minimum of stent diameter < 2.5 mm, ostium lesion, bifurcation lesion, LM lesion.

† Adjusted for comorbidities ( hypertension, DM uncontrolled, GI bleeding/ulcer, myocardial infarction) , use of aspirin, NSAID, multivessel PCI, number of stents, at least 1 DES, total stent length > 45 mm, minimum of stent diameter < 2.5 mm, ostium lesion, bifurcation lesion, LM lesion.

‡ Adjusted for age, comorbidities (hypertension, DM uncontrolled, GI bleeding/ulcer, peripheral vascular disease, myocardial infarction), use of aspirin, NSAID, multivessel PCI, number of stents, at least 1 DES, total stent length > 45 mm, minimum of stent diameter < 2.5 mm, ostium lesion, bifurcation lesion, LM lesion.

### The 12-months analysis

At 12 months after the index hospitalization, 45% (317/699) of patients were identified as continuous clopidogrel users. Compared to discontinuous clopidogrel users, continuous clopidogrel users were more likely to have a non-ST elevation myocardial infarction (NSTEMI) and DES implantation at index hospitalization. Continuous clopidogrel users had higher prevalence of hypertension, multi-vessel disease, right coronary artery (RCA) stenosis, longer stent length, and smaller stent diameter as well (Table 2).

Patients who received clopidogrel ≥ 12 months had a lower crude incidence rate of re-hospitalization for ACS (9.2%) compared to discontinuous group (13.9%). The clinical benefit of clopidogrel therapy ≥ 12 months after a mean follow-up of 2.2 years were statistically significant in the multivariable Cox regression models (risk for ACS re-hospitalization; adjusted HR 0.59, 95% CI 0.36-0.95; p = 0.03) (Table 3).

### The 15-months analysis

At 15 months after the index hospitalization, only 25% (164/653) of patients continued their clopidogrel use for more than 15 months. Compared to discontinuous users, patients received ≥ 15 months of clopidogrel therapy were older, had higher prevalence of multi-vessel disease and multiple stents, longer stent length, and were more likely to have a DES implantation during index hospitalization. Also, continuous users were more likely to have ostial lesion or left main (LM) artery stenosis. A 15-months of clopidogrel therapy was not associated with a significant reduction in ACS re-hospitalization (adjusted HR 0.57, 95% CI 0.29-1.13; p = 0.11) (Table 3).

### DES-subgroup analysis

Among patients who underwent at least one DES implantation, the risk of ACS re-hospitalization between patients who received ≥ 9 (N = 369) and <9 months of clopidogrel (N = 185) were not statistically different (adjusted HR 0.68; 95% CI 0.44-1.06; p = 0.09). In contrast, those who received clopidogrel for ≥ 12 months (N = 271), compared with those who discontinued within 12 months (N = 252), had a significantly lower risk of ACS re-hospitalization (adjusted HR 0.52; 95% CI 0.29-0.92; p = 0.02). However, the aforementioned clinical benefit was lost when clopidogrel therapy was extended for more than 15 months (N = 140) (adjusted HR 0.76; 95% CI 0.37-1.56; p = 0.45) (Table 4).

**Table 4:** Adjusted hazards for ACS re-hospitalization by clopidogrel duration (9, 12, 15 months); DES subgroup

**DES-subgroup analysis**	**Unadjusted hazard ratio**	p*	**Adjusted hazard ratio**	p*
	**HR (95% CI)**		**HR (95% CI)**	
Clopidogrel use for ≥ 9 mo	0.71 (0.47-1.07)	0.10	0.68 (0.44-1.06) ^*^	0.09
Clopidogrel use for ≥ 12 mo	0.58 (0.35-0.98)	0.04	0.52 (0.29-0.92) †	0.02
Clopidogrel use for ≥ 15 mo	0.91 (0.46-1.82)	0.79	0.76 (0.37-1.56) ‡	0.45

^*^Adjusted for comorbidities (hypertension, DM uncontrolled, myocardial infarction), use of aspirin, NSAID, multivessel PCI, number of stents, total stent length > 45 mm, minimum of stent diameter < 2.5 mm, ostium lesion, bifurcation lesion, LM lesion.

† Adjusted for comorbidities (hypertension, DM uncontrolled, GI bleeding/ulcer, myocardial infarction), use of aspirin, NSAID, cholesterol, multivessel PCI, number of stents, total stent length > 45 mm, minimum of stent diameter < 2.5 mm, ostium lesion, bifurcation lesion, LM lesion.

‡ Adjusted for comorbidities (hypertension, DM uncontrolled, GI bleeding/ulcer, myocardial infarction), use of NSAID, multivessel PCI, number of stents, total stent length > 45 mm, minimum of stent diameter < 2.5 mm, ostium lesion, bifurcation lesion, LM lesion.

### Other risk factors of ACS re-hospitalization

Other factors associated with ACS re-hospitalization in our multivariable Cox proportional models included multi-vessel disease (adjusted HR 1.77, 95% CI 1.12-2.80; p = 0.02), minimum of stent diameter < 2.5 mm (adjusted HR 3.19, 95% CI 1.29-7.88; p = 0.01), use of NSAID (adjusted HR 5.88, 95% CI 1.82-18.99; p = 0.003), and uncontrolled diabetes mellitus (adjusted HR 1.88, 95% CI 1.09-3.23; p = 0.02).

## Discussion

The main finding of this study is that there is significant benefit associated with ≥ 12 months of clopidogrel therapy in reducing the risk of ACS re-hospitalization among patients who underwent PCI for ACS. Our study provides further insights into clinical benefits of clopidogrel use beyond 12 months in this patient population. Nevertheless, clopidogrel therapy ≥ 15 months is not associated with significant clinical benefit in terms of reducing ACS re-hospitalization. Similar results are found in the DES population.

Our findings extend the current knowledge about the duration of clopidogrel use in a “real-world” ACS population. Conflicting data on the clinical benefits of continuous use of dual antiplatelet therapy between 6 months to 12 months have been reported in prior studies [12-15]. Kimura and his colleague [12] showed that continuous use of clopidogrel ≧ 6 months was not associated with a statistically significant higher risk of MI or death compared with continuous use of clopidogrel < 6 months (< 6 months 4.1% vs. ≧ 6 months 4.0%, p = 0.99). Park et al. [13] also found that the hazard ratios of stent thrombosis (HR 1.38; 95% CI 0.17-11.21; p = 0.77) and death or MI (HR 1.16; 95% CI 0.56-2.42; p = 0.69) were comparable among patients continuing and not continuing clopidogrel at 12 months. In contrast, the Clopidogrel for the Reduction of Events During Observation (CREDO) trial [14] suggested that at 1 year, long-term clopidogrel use was associated with a 26.9% relative reduction in the combined risk of death, MI, or stroke (95% CI 3.9%-44.4%; p = 0.02). Furthermore, an observational study done by Eisenstein et al. [15] found that discontinued clopidogrel before 12 months was associated with a higher risk of MI or death (4.5% vs. 0%) in the DES but not in the BMS group.

Along with several recent observational studies [16-18], our findings support the latest ACC /AHA guideline [6] that a at least 12-months of dual antiplatelet therapy following PCI would be beneficial to ACS patients. We found that, at a mean follow-up of approximately 2 years, 9 months of clopidogrel therapy only provided a marginal benefit in reducing the risk of ACS re-hospitalization (adjusted HR 0.69, 95% CI 0.48-1.00; p = 0.05) while 12 months of clopidogrel use was associated with a 40% reduction of the risk of ACS re-hospitalization (adjusted HR 0.59, 95% CI 0.36-0.95; p = 0.03). The 12 months of clopidogrel use is especially critical to the DES population. Among DES patients, we found a larger reduction in the hazard of ACS re-hospitalization (adjusted HR 0.52; 95% CI 0.29-0.92; p = 0.02) for patients received at least 12 months of clopidogrel but not 9 months of clopidogrel use.

Our study, however, does not support an extend duration of clopidogrel use to at least 15 months for patients who receive DES by the ACC /AHA guideline [6]. Our data shows that DES patients who received clopidogrel for at least 15 months was not associated with a decreased hazard of ACS re-hospitalization (adjusted HR 0.76; 95% CI 0.37-1.56; p = 0.45). To date, our study may be the first one to provide empirical evidence regarding the clinical benefit of extended clopidogrel to 15 months in ACS patients underwent PCI (especially DES). Given the increased bleeding risk and inherent economic burden with prolonged antiplatelet therapy [11,14], the most clinical and economic efficient strategy is to assure a 12 months of clopidogrel to all ACS patients who undergo PCI until we have more evidence.

There are inherent limitations with our retrospective observational study design. As with any non-randomized design, significant differences in comorbidities between patients who continued clopidogrel or not could have affected hazard estimations of ACS re-hospitalization. However, our patients in the continuous and discontinuous groups were well balanced in terms of most baseline characteristics, including prevalence of peripheral vascular diseases. In addition, our hospital-based setting also allowed us to adjust for a wide range of potential cardiovascular risk factors, including very detailed procedural characteristics and information on other concomitant medications (such as ACEI/ARBs, lipid-lowering agents, and NSAID). Second, because of its retrospective nature, we do not know the reasons for clopidogrel discontinuation. Thirdly, our findings may not be generalizable to non-Asian populations. Previous study has indicated that the cytochrome P450 (CYP) 2C19 poor metabolizers may exhibit less antiplatelet activity when they were exposed to the same clopidogrel regimen than other healthy volunteers. The variation of prevalence of CYP2C19 poor metabolizers in different populations (3-6% of Europeans and Africans, and 13-23% of Asians) thus need to be taken into account when interpreting our findings [19]. Fourthly, we did not report risk of bleeding according to the duration of clopidogrel as it was not the main focus of our study. Fifthly, we did not include newer antiplatelet agents such as prasugrel and ticagrelor in our study as both of them were not reimbursed by Taiwan’s NHI program during our study period (prasugrel: currently not available; ticagrelor (available since July, 2013)).

Despite these limitations, this study has several significant methodological strengths. First, the medication utilization data were based both on the hospital electronic pharmacy prescription database and medical charts. Previous observational studies, however, relied on pharmacy claims [17,20] and seldom captured clopidogrel use paid out-of-pocket. Furthermore, because aspirin prescriptions are covered by the NHI program, patients seldom obtain aspirin over the counter. This allows us to capture the “dual” antiplatelet therapy in our study cohort. For example, at 9 months of follow-up, approximately 87% of the discontinuous users and 80% of the continuous users concomitantly used aspirin. Another methodological strength with the hospital-based data source is that we could include very detailed procedural characteristics in our model to provide better estimations of hazards of ACS re-hospitalization.

## Conclusion

Our study provides new evidence on the minimal necessary clopidogrel use in an ACS population. Together, our study and findings of recently published studies support the latest clinical guideline that a 12-months of clopidogrel therapy conveys an important clinical benefit after PCI (especially DES). Further studies are required to evaluate the clinical benefits of clopidogrel use up to 15 months or more.

## Abbreviations

ACS: Acute coronary syndrome; PCI: Percutaneous coronary intervention; DES: Drug-eluting stents; CABG: Coronary artery bypass graft; STEMI: ST-elevation myocardial infarction; NSTEMI: Non-ST elevation myocardial infarction.

## Competing interest

The authors declare that they have no competing interest.

## Authors’ contribution

HFY, LCM, GCS, and CSC were responsible for development of the study concept and design, and for the preparation of the manuscript. CSC contributed to the data acquisition and statistical analysis. HWWY contributed to the revision of the manuscript. All authors participated in the analysis and interpretation of the data, read and approved the manuscript for submission.

## Pre-publication history

The pre-publication history for this paper can be accessed here:

http://www.biomedcentral.com/1471-2261/14/21/prepub
